# Characterization of Asphalt Binder Properties Modified with One-Time Use Masks: Zero Shear Viscosity, Fatigue Life, and Low-Temperature Performance

**DOI:** 10.3390/ma18214861

**Published:** 2025-10-23

**Authors:** Alaaeldin A. A. Abdelmagid, Guanghui Jin, Guocan Chen, Nauman Ijaz, Baotao Huang, Yiming Li, Aboubaker I. B. Idriss

**Affiliations:** 1 School of Civil Engineering, Quanzhou University of Information Engineering, Quanzhou 362000, China; alaaeldin@qzuie.edu.cn (A.A.A.A.); cgc@qzuie.edu.cn (G.C.); nauman_ijaz@qzuie.edu.cn (N.I.); 2 School of Civil and Transportation Engineering, Northeast Forestry University, Harbin 150040, China; jin1650@nefu.edu.cn; 3 College of Mechanical and Electrical Engineering, Northeast Forestry University, Harbin 150040, China; 4 Department of Mechanical Engineering, Faculty of Engineering Science, University of Nyala, P.O. Box 155, Nyala 62712, Sudan

**Keywords:** one-time use masks, modified asphalt, linear amplitude sweep, zero shear viscosity, low temperature performance

## Abstract

The widespread adoption of one-time use masks (OUM) has resulted in a substantial new stream of polymer waste, posing a formidable challenge to circular economy and waste management initiatives. Concurrently, the pavement industry continuously seeks innovative modifiers to enhance the durability and service life of asphalt binders. This study presents a novel approach to waste valorization by systematically investigating the potential of shredded OUM as a polymer modifier for asphalt. The research evaluates the impact of various OUM concentrations (up to 10% by weight) on the binder’s chemical, rheological, and performance characteristics. Fourier-transform infrared spectroscopy (FTIR) indicated that the modification is a physical blending process, with the OUM fibers forming a stable reinforcing network within the asphalt matrix, a finding supported by excellent high-temperature storage stability. Rheological assessments revealed a remarkable enhancement in high-temperature performance, with the Zero-Shear Viscosity (ZSV) increasing by nearly 700% (from approximately 450 Pa·s to about 3500 Pa·s) at 10% OUM content, signifying superior rutting resistance. Furthermore, fatigue life, evaluated via the Linear Amplitude Sweep (LAS) test, improved by up to 168% at a 2.5% strain level. However, these benefits were accompanied by a detrimental effect on low-temperature properties, where creep stiffness at −12 °C increased by over 50% and the m-value dropped below the critical 0.30 threshold, indicating a heightened risk of thermal cracking. The study concludes that OUM is a highly effective modifier for improving high-temperature and fatigue performance, with up to 10% content being viable. This research establishes a promising circular economy pathway, transforming a problematic waste stream into a valuable resource for constructing more resilient and sustainable pavement infrastructure.

## 1. Introduction

Following the emergence of the COVID-19 pandemic, the practice of wearing masks shifted from a provisional safety measure to a firmly established daily habit. This change is largely attributed to several factors, including government regulations, personal risk evaluations, and the pervasive influence of the media [1,2]. Mask usage, first implemented as a critical health intervention, remains widespread even though the acute threat posed by the virus has lessened, signaling permanent changes in community practices [3]. Frequent mask usage, supported by a subjective feeling of security and the motivation to prevent possible health threats, has transformed mask-wearing into a habitual behaviour [4]. Nevertheless, the extensive use of masks has generated considerable ecological and financial challenges, particularly related to their disposal. Inadequate handling of used masks generates substantial difficulties in waste management, thereby affecting ecological systems and the wider economic landscape [5,6,7]. When face masks are discarded improperly, they tend to accumulate in aquatic environments such as rivers and seas, presenting considerable dangers to aquatic species [8]. According to recent analyses, mask debris ranging from 0.15 to 0.39 million tons is dumped into the ocean annually, which putting ocean ecosystems at risk [9]. The management of mask waste through traditional techniques, such as thermal incineration at high temperatures, causes twofold environmental consequences. Both aggravating climate change via greenhouse gas emissions and emitting harmful toxins into the atmosphere, these processes collectively heighten environmental pollution [10]. The practice of dumping used masks in waste disposal sites is impractical, as it poses a risk to soil quality through contamination [11,12]. In addition, the slow degradation of masks, which can take centuries, leads to long-lasting environmental concerns [13]. To tackle this issue, a viable strategy is to incorporate discarded mask materials into asphalt, thereby facilitating the development of more environmentally friendly infrastructure projects. By transforming this problematic waste into a high-performance modifier, this approach offers a potential dual benefit: reducing pollution and creating a cost-effective alternative to virgin polymers. For clarity, it should be noted that in this study, the term ‘asphalt’ refers specifically to the ‘asphalt binder’ (also known as bitumen), which is the viscoelastic liquid that binds the aggregates in an asphalt mixture.

The composition of one-time use masks (OUM) includes several polymeric substances such as polyester and polypropylene (PP) [14]. Among them, PP represents the principal ingredient [15]. Because of its considerable molecular mass, PP is an effective additive for asphalt and its mixture. Investigations have consistently revealed that asphalt modified with PP demonstrates greater properties compared to the original asphalt [16,17]. Additionally, the integration of PP enhances key properties of asphalt, including its deformation resistance, flow behavior, and thermal durability [18,19]. An asphalt formulation integrating waste organic rectorite (1.5%) and PP (4%) was designed by Chen and co-authors, yielding a modified material with increased deformability and ductility [20]. In addition, the presence of PP within asphalt composites contributed to strengthening their indirect tensile strength, Marshall performance, and deformation durability [21,22]. In comparison with the unmodified mixture, the incorporation of PP into the asphalt resulted in corresponding enhancements of 67.5%, 38%, and 26.3% in void ratio, workability assessment, and Marshall strength [22]. Research by Wang et al. indicates that binder composites strengthened by PP fibers exhibited enhanced crack resistance and higher load-bearing capacity relative to compositions incorporating polyester and various fibers [23]. Adding SBR and PP to binder increased its wear resistance and decreased its rutting, according to another study [24]. In addition, evidence suggests that polypropylene-enhanced asphalt can lessen the damage to pavement induced by wear [25]. Moreover, PP compounds are utilized to mitigate elevated pavement surface temperatures in summertime [26]. However, despite these demonstrated advantages, the practical implementation of polymer-modified binders (PMBs), particularly those utilizing recycled or waste polymers, is fraught with significant challenges that have hindered their widespread adoption [27,28,29]. A primary obstacle is the inherent incompatibility between many polymers and the asphalt matrix, which arises from differences in their chemical structure and polarity. This often leads to phase separation during high-temperature storage, resulting in a non-homogeneous binder with compromised mechanical properties and a loss of the intended rheological benefits [27,28]. This issue is often exacerbated by feedstock variability, especially with recycled plastics, where inconsistent polymer type, molecular weight, and impurity content can lead to unpredictable performance outcomes [29].

Furthermore, achieving a stable, homogeneous blend presents another major hurdle, often requiring precisely controlled high-shear mixing at elevated temperatures for extended durations [30,31]. Such processing demands can be energy-intensive and difficult to replicate consistently at the field scale. The performance of many polymer-modified systems is also highly temperature-dependent; while they typically improve high-temperature stiffness, they can inadvertently embrittle the asphalt at low service temperatures, thereby increasing cracking potential [32,33]. This performance can be further degraded over time by aging and continued phase separation during the pavement’s service life [34,35]. Collectively, these technical challenges contribute to implementation and economic barriers, including a lack of universal standards and uncertainties in long-term durability, which can slow field adoption despite the clear potential of the technology [36,37].

Asphalt pavements commonly exhibit rutting, which is identified as a significant mode of deterioration. Presently, the combined influence of applied loads and thermal stresses renders these surfaces vulnerable to lasting deformation [38]. Furthermore, delayed maintenance leads to an increasing severity of deformation, which may cause significant harm to the pavement structure over time. Consequently, the functionality of the road deteriorates, thereby hastening the reduction in the asphalt pavement’s lifespan. This circumstance negatively impacts both economic performance and environmental sustainability [39]. To evaluate asphalt binders’ resistance to rutting, the Superpave framework measures their elasticity and stiffness by using a dynamic shear rheometer (DSR) under intermediate and high-temperature conditions. Rutting resistance was assessed using the rutting indicator (G*/sin δ). Multiple laboratory investigations have employed this factor to assess the rutting behaviour of both unmodified and modified asphalt binders, utilizing a DSR to simulate real-world conditions [40,41,42,43]. Research has demonstrated that the G*/sin δ parameter is not a reliable indicator when evaluating the rutting behavior of modified asphalt binders [44,45]. Zero shear viscosity (ZSV) has emerged as a sophisticated technique for evaluating the rutting resistance of asphalt binders, drawing substantial interest from researchers in recent years [46,47]. The viscosity of polymer-modified bitumen decreases with increasing shear rate, reflecting its typical non-Newtonian fluid behavior at elevated temperatures [48]. Research has revealed that the viscosity of asphalt tends to reach a consistent value when subjected to either minimal or extremely high shear rates. Consequently, ZSV was proposed as an evaluation metric to characterize the rutting resistance of asphalt binders, especially those modified with polymers, thereby minimizing the impact of shear rate on test outcomes [40,49,50]. Accordingly, in the present investigation, ZSV was utilized to evaluate the thermal behaviour of base asphalt and its modified counterpart at elevated temperatures.

An additional critical challenge faced by asphalt pavements is fatigue damage. The fatigue behaviour of asphalt binders was investigated using the Superpave fatigue parameter (G* sin δ) [51,52]. Nevertheless, this metric fails to account for the actual physical damage sustained by the asphalt binder because the measurement is conducted within the linear viscoelastic regime [53,54,55]. In recent years, innovative fatigue assessment techniques, including the linear amplitude sweep (LAS), have attracted significant interest from the research community, owing to the fact that it offers an improved and accelerated procedure for measuring fatigue resistance [56,57]. Damage resistance of the binder is evaluated in the LAS test through repetitive loading where the load amplitudes increase in a linear way. This method employs the viscoelastic continuum damage (VECD) theory [58,59]. The rate at which damage accumulates reflects the fatigue behaviour of the binder. The procedure’s validation was achieved by evaluating the correlation between mixture fatigue performance and practical results observed in the field [60,61]. Consequently, LAS was used in this investigation to evaluate the fatigue resistance exhibited by both the unmodified asphalt and its modified form.

The low-temperature cracking resistance of asphalt binders is a critical factor in ensuring the durability and longevity of asphalt pavements [62,63]. The bending beam rheometer (BBR) serves as a widely utilized instrument for evaluating asphalt binders by quantifying their creep stiffness and m-value [64]. The test evaluates the stiffness and viscoelastic relaxation characteristics of bituminous binders under cold conditions.

Grounded in previous research, this investigation is driven by the urgent need to tackle both the deteriorating effectiveness of conventional asphalt and the environmental challenges resulting from improper disposal of OUM. While the use of polymers to modify asphalt is well-established, this study focuses on the specific application of waste OUMs as a functional modifier. The innovation, therefore, lies not in the creation of a new polymer modifier, but in providing a practical and effective recycling pathway for a problematic waste stream. Accordingly, the present research is designed to rigorously examine how different proportions of OUM influence the performance of binders across at elevated, moderate, and low temperatures, employing experimental lab methods. The key aims have been defined as outlined below:

1. To scrutinize the effects of OUM inclusion on the ZSV of asphalt binders by means of steady shear tests.
2. To examine the influence of OUM integration within asphalt binder on fatigue performance through the application of the LAS test.
3. Using bending beam rheometer methods, this research explores the low-temperature cracking performance of OUM-modified binders (OUMM) through assessment of creep rate and stiffness parameters.
4. Utilizing Fourier transform infrared (FTIR) analysis for assessing the influence of OUM application on the functional groups of asphalt samples.
5. To explore the impact of OUM on asphalt binder’s storage stability through application of the separation test method.

While the use of waste polymers in asphalt is well-documented, the specific application of waste medical masks has recently emerged as a significant area of interest. Initial studies have successfully demonstrated that incorporating shredded masks can enhance the mechanical properties and rutting resistance of asphalt mixtures [65,66,67]. However, a gap remains in the literature for a systematic characterization of OUM-modified binders across a wide performance spectrum using advanced, mechanism-based testing protocols. Prior research has largely focused on conventional mix properties without a deep, binder-level investigation into the complex rheological trade-offs. The novelty of this study lies in its rigorous and integrated evaluation, ZSV to define rutting resistance, LAS test to quantify fatigue life, and BBR tests to precisely assess low-temperature behavior. By systematically quantifying the critical performance balance for the first time detailing not only the significant improvements in high and intermediate-temperature properties but also the concurrent, pronounced negative impact on low-temperature performance, this research provides an essential, data-driven assessment. This work establishes a foundational dataset for a circular economy approach, demonstrating a viable pathway, and its precise limitations, for converting this problematic waste stream into a high-value resource for pavement engineering.

**Figure 1:**
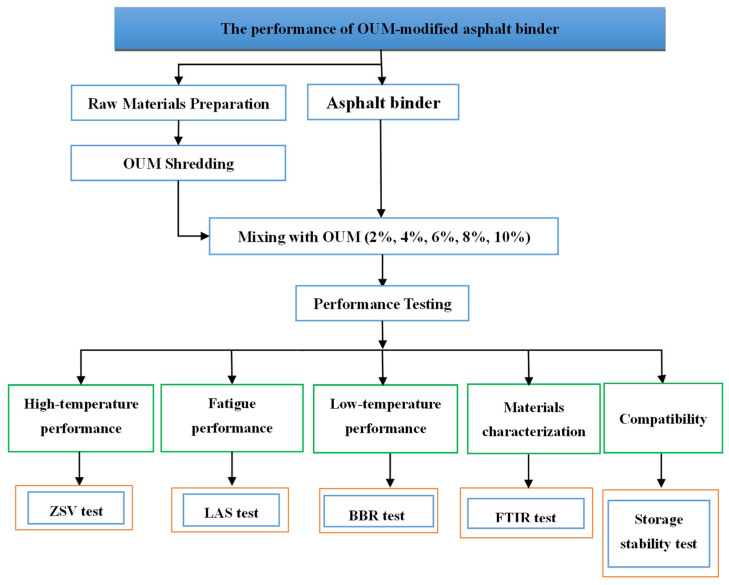
Experimental scheme.

Figure 1 presents a schematic flowchart of the experimental program, outlining the key stages from material preparation to final performance testing.

## 2. Materials and Test Program

### 2.1. Raw Materials

The present study employed 60/80-graded asphalt procured from China Petroleum & Chemical Corporation, Beijing, China, with the relevant physical characteristics exhibited in Table 1. Assessment of these characteristics was carried out in line with the 60/80 penetration grade criteria detailed in JTG F40 2004.

It is critical to acknowledge that the use of unutilized OUMs in this study serves to establish a controlled scientific baseline. This approach was intentionally chosen to eliminate the confounding effects of contaminants and material degradation inherent in post-consumer waste, thereby allowing for an unambiguous characterization of the PP fibers’ direct impact on asphalt binder properties. However, for the practical, large-scale application of this technology, the processing of actual post-consumer waste masks is a prerequisite. This would involve a multi-stage pre-treatment process. First, a robust sterilization protocol is necessary to neutralize biological hazards. Potential methods include autoclaving, which has been shown to be effective, though care must be taken to ensure temperatures do not significantly degrade the PP polymer. Alternative non-thermal methods like ultraviolet germicidal irradiation (UVGI) or chemical disinfection using ozone or peracetic acid could also be employed. Second, mechanical separation of the non-polymeric components is required. The metallic nose strips can be efficiently removed using magnetic separators following initial shredding. The cotton or elastic ear loops, which have a different density and texture from the PP fabric, could be separated using advanced sorting technologies such as air classification or float-sink separation methods. The successful implementation of these pre-treatment steps is essential for transforming post-consumer mask waste into a safe and consistent feedstock for asphalt modification, forming a crucial area for future pilot-scale research.

**Figure 2:**
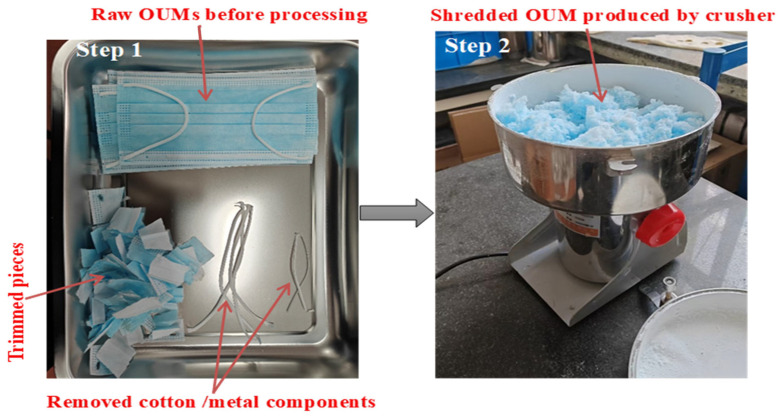
Preparation and shredding of OUM prior to experimentation.

Unutilized OUM was employed for assessment, with the relevant physical characteristics shown in Table 2. Before the experimentation, the OUM’s metallic and cotton components were eliminated. The cleaned OUM was then trimmed into sections roughly 0.03 m in width and 0.05 m in length, and these OUM pieces were subsequently processed in a crusher to yield shredded OUM, which is depicted in Figure 2.

The crushing process resulted in a fibrous, fluffy material rather than granular particles. While a quantitative particle size distribution was not performed, the subsequent high-shear mixing process (Section 2.2) is designed to ensure thorough dispersion of these fibers. The uniformity and stability of this dispersion were subsequently confirmed by the storage stability test (Section 3.5), where the minimal difference in softening points between the top and bottom sections of the stored samples indicated that a homogeneous and stable mixture was achieved.

### 2.2. OUMM Preparation

**Figure 3:**
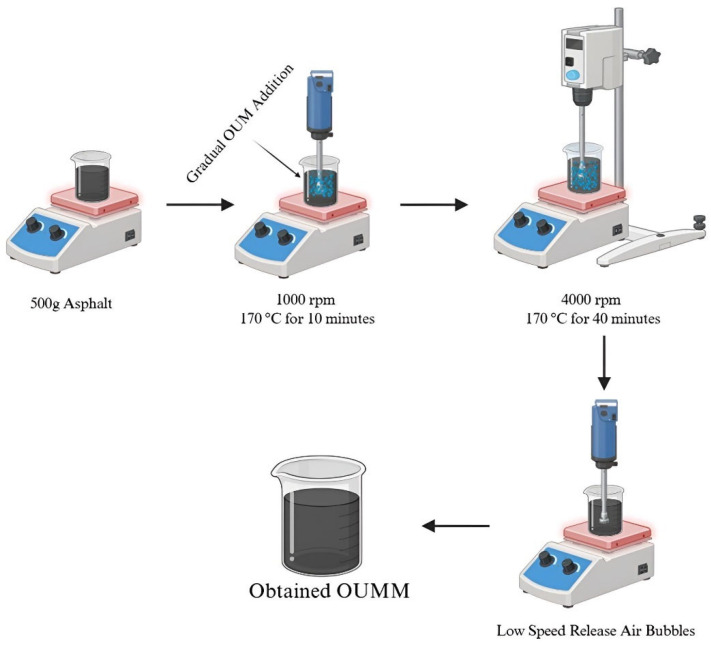
OUMM preparation.

Samples in this study were prepared employing high-shear as well as mechanical mixers. The study utilized five varying OUM concentrations 2%, 4%, 6%, 8%, and 10% in terms of their weight compared to the asphalt. Initially, 500 g of base asphalt was liquefied by heating, and OUM was slowly incorporated in measured increments. The mixture was mechanically mixed at 1000 rpm for 10 min before being exposed to high-shear mixing at 4000 rpm and 170 °C for 40 min [68]. For the purpose of expelling any remaining air, the compound was stirred gently using a mechanical mixer set to a low speed for ten minutes, as demonstrated in Figure 3.

### 2.3. High-Temperature Performance

**Figure 4:**
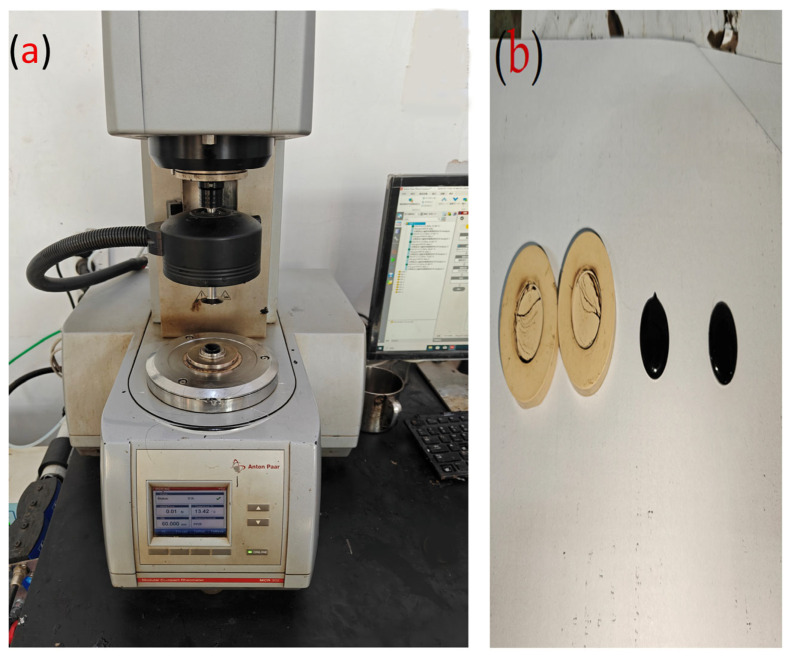
(**a**) DSR device, and (**b**) Asphalt binder sample.

The ZSV of asphalt binder demonstrates a strong association with its capacity to resist rutting, thereby serving as a reliable parameter for evaluating performance at elevated temperatures. This ZSV, defined as the viscosity at nearly zero shear rate (η_0_) [69], was determined via steady-state flow testing using a DSR device (Anton Paar, Graz, Austria) (Figure 4a) on binder samples (0.025 m diameter, 0.001 m thickness, Figure 4b), at 60 °C. Figure 4a presents the MCR302 Dynamic Shear Rheometer (Anton Paar, Graz, Austria) utilized for evaluating the high-temperature rheological characteristics of both unmodified and OUMM. This precision rheometer employs a parallel plate geometry (25 mm diameter, 1 mm gap) to subject specimens to controlled shear deformation under isothermal conditions at 60 °C. The instrument measures the phase angle (δ) and complex shear modulus (G*), enabling determination of the zero-shear viscosity through steady-state flow testing across shear rates ranging from 0.01 to 100 s^−1^. The DSR’s capability to characterize non-Newtonian flow behavior is critical for understanding the polymer network formation within the OUMM which manifests as pronounced shear-thinning characteristics indicative of successful binder modification. In Equation (1) [70], the Carreau model is mathematically represented.

(1) $$\begin{array}{cccc} & \eta =\eta_{\infty }+\frac{\eta_{0}-\eta_{\infty }}{{\left(1+{\left(\frac{\gamma }{\gamma_{c}}\right)}^{2}\right)}^{s}} & & \end{array}$$

Within this formulation, *s* reflects the parameter associated with the gradient of the shear-thinning segment, $\dot{\gamma_{c}}$ designates the shear rate where shear thinning commences, *η_∞_* is the viscosity limit at infinite shear rate, *η_0_* stands for the viscosity in the zero-shear condition, and *η* describes the viscosity under varying shear rates.

### 2.4. Fatigue Life Assessment

The assessment of asphalt’s fatigue durability under repetitive load is commonly achieved through the LAS method. According to AASHTO TP101 and utilizing the DSR apparatus, the LAS encompasses a frequency sweep ranging from 0 to 30 Hz at 0.1% strain, alongside an amplitude sweep, which is implemented at a frequency of 10 Hz. The present study examined the fatigue behavior of asphalt at a temperature of 25 °C. For testing, positioning the material between two test plates of 8 mm dimension, separated by a constant 2 mm gap. The durability of binder specimens against fatigue was quantified using continuum damage theory.

Initially, a plot was created with the logarithm of the storage modulus on the y-axis and the logarithm of the imposed frequency on the x-axis, which produced a linear trend based on the frequency sweep data. The gradient of this line (m) was then determined, and the relevant parameter was computed as its reciprocal. Subsequently, the accumulated damage within the specimen was calculated using the following equation:

(2) $$\begin{array}{cccc} & D(t)≅\sum_{i=1}^{N}{\left[\pi \gamma_{0}^{2}(C_{i-1}-C_{i}\right]}^{\alpha /(1+\alpha )}(t_{i}-t_{i-1}{)}^{1/(1+\alpha )} & & \end{array}$$

where D(t) refers to the cumulative damage within the sample; *C_i_* denotes the proportion of the complex modulus at time t relative to its initial value, expressed as G*(t)/G*(initial); *γ*_0_ represents the imposed strain magnitude (as a percentage); and *α* corresponds to the reciprocal of the gradient obtained from the plot of the logarithmic storage modulus against the logarithmic applied frequency.

A power law model is employed to characterize the association between C(t) and D(t) as follows:

(3) $$\begin{array}{cccc} & C_{\left(t\right)}=C_{0}-C_{1}(D{)}^{C_{2}} & & \end{array}$$

The integrity of the specimen is represented by *C_(t)_*, with *C_0_*, *C_1_*, and *C_2_* acting as parameters obtained through curve fitting. The calculation of failure point D_f_ is based on the value of C at its peak shear stress, as prescribed by the following equation:

(4) $$\begin{array}{cccc} & D_{f}={\left(\frac{C_{0}-C_{atpeakstress}}{C_{1}}\right)}^{1/C_{2}} & & \end{array}$$

The fatigue life in terms of cycles to failure, denoted as *N_f_*, is obtained by means of the following mathematical expression:

(5) $$\begin{array}{cccc} & N_{f}=A\;(\gamma_{max}{)}^{-B} & & \end{array}$$

Within the above formula, *A* and *B* are coefficients obtained via regression fitting, and *γ*_*max*_ represents the maximum strain level.

(6) $$\begin{array}{cccc} & A=\frac{f(D_{f}{)}^{1+(1-C_{2})\alpha }}{(1+(1-C_{2})\alpha )\times (\pi C_{1}C_{2})\alpha } & & \end{array}$$

(7) $$\begin{array}{cccc} & B=2\alpha & & \end{array}$$

Here, k is expressed by the relation k = 1 + (1 − *C_2_*), and f denotes a load frequency of 10 Hz.

### 2.5. Low-Temperature Performance

**Figure 5:**
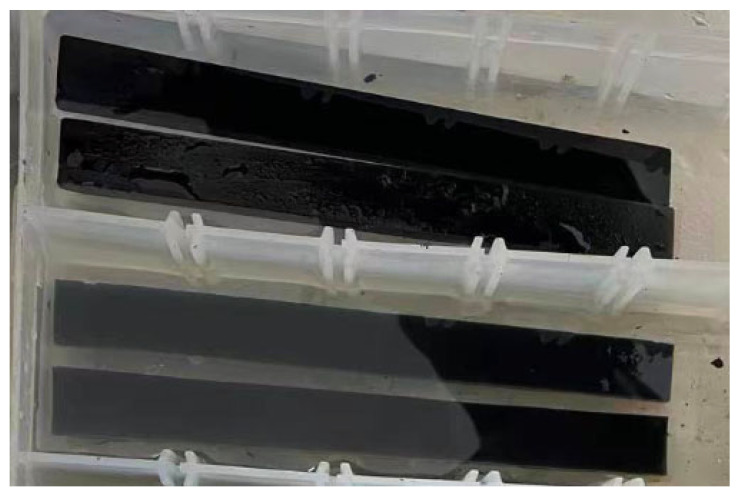
BBR samples.

The BBR procedure, implemented according to the AASHTO T313-12 standard, examined the asphalt binder’s tendency to crack in cold climates. This testing approach focuses on the measurement of creep stiffness and creep rate, both critical for assessing the binder’s performance against low-temperature cracking. The experiment was conducted at temperatures of −6 °C, −12 °C, and −18 °C, utilizing binder beam specimens shaped as rectangles (Figure 5), each measuring 0.125 m in length, 0.0125 m in width, and 0.00635 m in thickness. All beam samples underwent loading at a constant force of 0.98 N over a 240 s period. Center-point deflections were measured at designated time intervals (8, 15, 30, 60, 120, and 240 s) and mapped as a function of time. The m-value representing creep rate and the S value for creep stiffness at 60 s were calculated and subjected to further analysis.

### 2.6. FTIR Test

FTIR spectroscopy was employed as the primary analytical tool to verify the chemical nature of the modification process. The objective was to confirm the physical blending of the polypropylene from the OUM into the asphalt matrix and to detect any potential chemical degradation or interaction by tracking the characteristic functional groups of both components. In this study, measurements were conducted in the 4000–500 cm^−1^ wavenumber range using a Nicolet iS 50 FTIR spectrometer (Thermo Fisher Scientific, Waltham, MA, USA). To prepare the samples, binders were dissolved in carbon disulfide to achieve a 5 wt.% solution, then deposited onto potassium bromide windows and dried prior to analysis.

### 2.7. Storage Stability Test

To evaluate the stability of the OUMM when stored at high temperatures, a storage stability test was performed. Fifty grams of asphalt were loaded into an aluminum tube measuring 0.14 m long and 0.025 m wide. The tube was heated at 163 °C for 48 h and then cooled for 4 h at −4 °C. The tube was then sectioned into three equal parts, and stability was scrutinized by comparing the softening point differences between samples taken from the top and bottom portions.

## 3. Results and Discussion

### 3.1. ZSV Results

**Figure 6:**
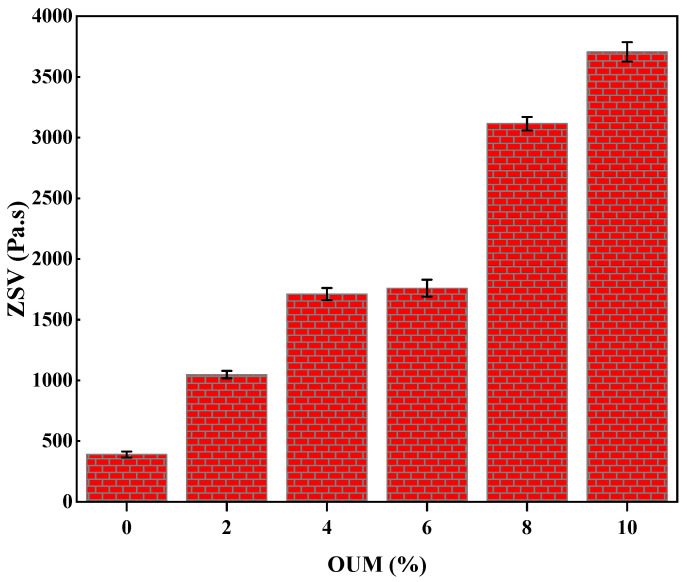
ZSV values at various OUMM concentrations.

The incorporation of OUM fundamentally transformed the high-temperature rheological characteristics of asphalt binder, a critical determinant of pavement resistance to permanent deformation. As illustrated in Figure 6, the ZSV as a key indicator of a material’s resistance to flow under terminal conditions, underwent a substantial and systematic augmentation with increasing OUM content. This phenomenon is rooted in the physical interactions between the asphalt binder and the PP polymer from the OUM. As confirmed by FTIR analysis which showed no chemical changes, the modification process involves the absorption of the asphalt’s lighter aromatic fractions (maltenes) by the PP chains. This causes the polymer to swell, forming an intricate, three-dimensional physical network [71]. This network structure, evidenced by the dramatic increase in viscosity, creates significant steric hindrance and a high density of entanglement points, which severely restricts the motion of the asphalt molecules [72]. Consequently, the energy required to induce flow is significantly increased, resulting in a dramatic rise in ZSV from below 500 Pa·s for the base asphalt to above 3500 Pa·s at 10% OUM. This enhancement of nearly an order of magnitude signifies a fundamental improvement in the material’s rutting resistance, a correlation well-established in asphalt rheology [73].

**Figure 7:**
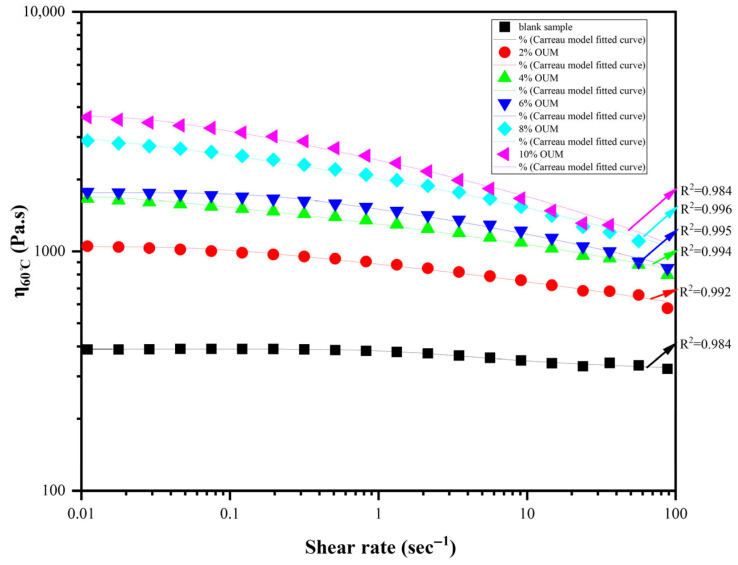
Model fitted curve.

Beyond static resistance, the dynamic flow behavior of the modified binders, depicted in Figure 7, reveals a crucial transition from a simple viscous fluid to a complex, structured non-Newtonian system. While the base asphalt exhibits near-Newtonian behavior, the OUMM displays a pronounced shear-thinning rheological fingerprint, a classic hallmark of polymer-modified asphalts [74]. This behavior is a direct consequence of the polymer network’s response to external shear stress. At low shear rates, the swollen polymer chains exist in a randomly oriented, high-entropy coil conformation, maximizing intermolecular friction and resulting in the high viscosity observed in the ZSV. As the shear rate increases, the applied stress overcomes the network’s relaxation time, forcing the entangled chains to disentangle, uncoil, and align in the direction of flow [71]. This shear-induced anisotropy drastically reduces the hydrodynamic volume and internal flow resistance, causing the observed decrease in viscosity. The Carreau model, chosen for its ability to accurately capture this entire flow curve from the low-shear Newtonian plateau to the power-law region exhibited an excellent fit to the empirical data for all modified binders (R^2^ > 0.98), quantitatively validating this mechanistic interpretation.

From a practical engineering standpoint, this shear-thinning characteristic is highly advantageous. It ensures lower viscosity at high shear rates (e.g., during plant mixing and field compaction) for excellent workability, while providing the high viscosity at low shear rates necessary for structural integrity and resistance to deformation under traffic loading. Accordingly, the incorporation of OUM waste operates as a functional modifier rather than inert filler, actively tailoring the binder’s rheological response and yielding demonstrably superior performance.

The temperature susceptibility of ZSV in polymer-modified asphalts is a critical performance consideration. While this study focused on 60 °C as the standard high-temperature evaluation point, established rheological theory predicts that OUMM would exhibit reduced temperature susceptibility compared to neat asphalt. Polymer networks, such as those formed by PP fibers from OUM, maintain structural integrity across broader temperature ranges than unmodified binders, providing a ‘reinforcement effect’ that persists even as asphalt viscosity decreases with temperature. At 50 °C, ZSV values would be expected to increase by approximately one order of magnitude for both neat and modified binders due to reduced molecular mobility, while at 70 °C, a corresponding decrease would occur. Critically, the ratio of modified-to-unmodified ZSV likely remains favorable across this temperature range, suggesting that OUMM’s rutting resistance advantage persists under varying climatic conditions. The physical modification mechanism confirmed by FTIR analysis, polymer network formation without chemical bonding, typically produces less temperature-sensitive behavior than neat asphalt’s purely viscous response. Future research incorporating multi-temperature ZSV evaluation would provide valuable quantification of OUMM’s temperature susceptibility coefficient for refined performance prediction across diverse geographic regions.

The substantial increase in ZSV observed in this study is consistent with the findings of other research on PP and waste mask-modified binders. The formation of a swollen polymer network that restricts the movement of asphalt molecules is a well-established mechanism for improving high-temperature performance. Yang et al. [75] reviewed various polymer modifiers and noted that PP effectively enhances binder stiffness and rutting resistance. More specifically, recent studies using waste masks have also reported significant improvements in high-temperature properties. For example, Abdelmagid et al. [68] reported similar enhancements in high-temperature performance when using single-use masks. Our finding of a nearly 700% increase in ZSV at 10% OUM content provides a quantitative benchmark that confirms OUM’s efficacy as a high-performance rutting-resistant modifier, placing its effectiveness in a range comparable to or exceeding some conventional polymers.

### 3.2. LAS Results

**Figure 8:**
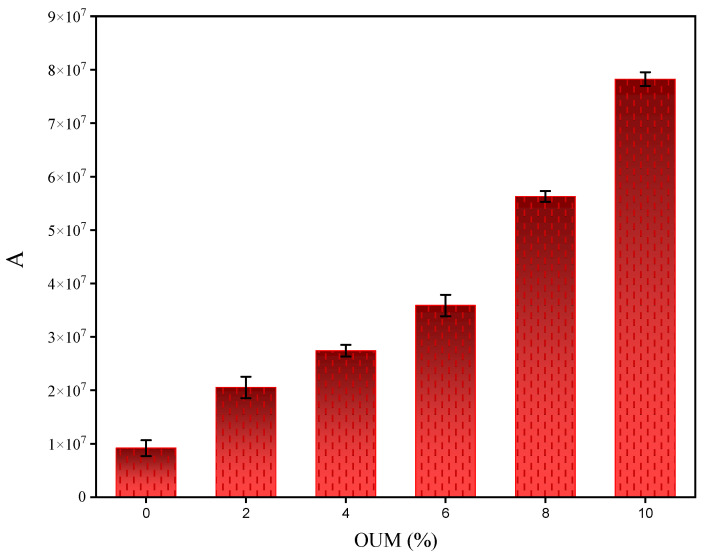
Influence of OUM on Fatigue Life Parameter (A).

**Figure 9:**
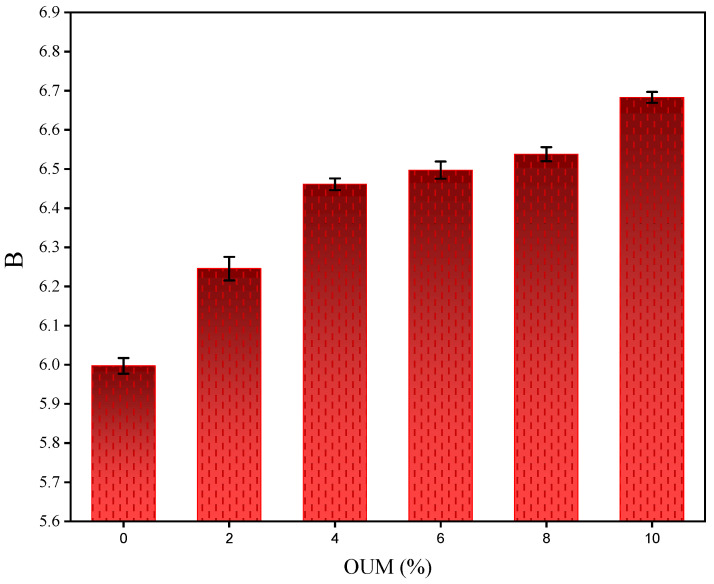
Influence of OUM on Strain Sensitivity Parameter (B).

The fatigue resistance of the asphalt binders modified with varying percentages of OUM was evaluated using the LAS test, with the key performance parameters, A and B [76], derived from the VECD model, presented in Figure 8 and Figure 9. Parameter A correlates with the storage modulus G* cos δ of the binders and is employed to assess the integrity of the material subjected to cumulative damage throughout the loading phase. An elevated A value reflects enhanced fatigue resistance. Conversely, Parameter B illustrates the material’s responsiveness to different load intensities, where a lower value indicates diminished damage progression across diverse strain magnitude.

The results indicate a substantial and systematic improvement in fatigue performance with the increasing addition of OUM. As shown in Figure 8, parameter A, which is directly proportional to the binder’s fatigue life, exhibits a pronounced monotonic increase. The value raises from approximately 1.0 × 10^7^ for the base asphalt to a remarkable 7.9 × 10^7^ for the binder containing 10% OUM, signifying an almost eight-fold enhancement in the predicted number of cycles to failure. This significant improvement can be attributed to the formation of a robust polymer network within the asphalt matrix by the PP from the OUM. This network acts as a reinforcing phase, enhancing the binder’s elasticity and its ability to dissipate strain energy, thereby effectively impeding the initiation and propagation of micro-cracks under cyclic loading [77].

Concurrently, Figure 9 illustrates that parameter B, which represents the sensitivity of fatigue life to the applied strain amplitude, also demonstrates a consistent increase from approximately 6.0 to 6.7. A higher B value signifies a greater rate of damage accumulation per loading cycle as strain increases. Therefore, the increase in parameter B suggests that while the stiffer and more elastic OUM-modified binder is superior at resisting damage initiation (high A value), its post-initiation response is more sensitive to strain. This indicates that once damage has commenced, it may propagate more rapidly at higher strain levels, a behavior that can be described as more brittle-like in its failure progression compared to the more ductile failure of the base binder. This leads to a greater rate of damage accumulation per cycle at a given strain, hence the higher B value.

The outcome reveals that while OUMM maintains enhanced structural robustness during cyclic loading, its fatigue lifespan shortens at a quicker rate with rising strain amplitudes.

**Figure 10:**
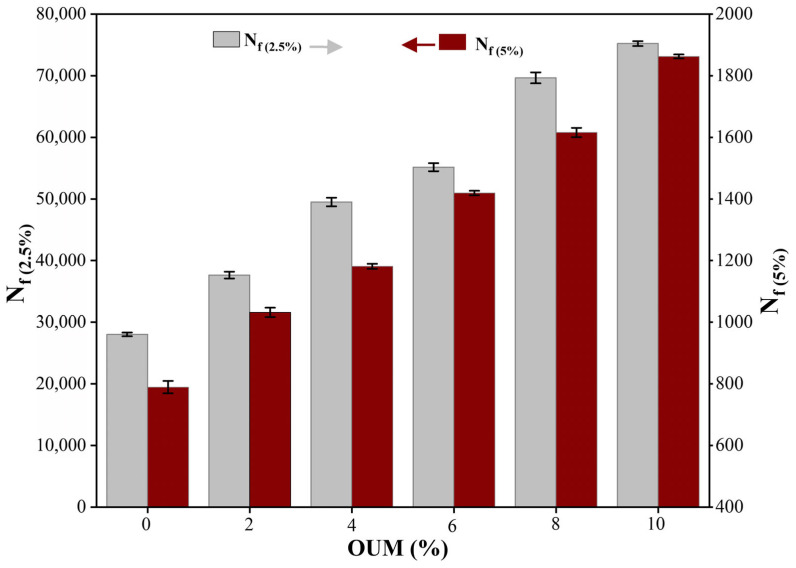
The fatigue life at different OUM content.

Figure 10 illustrates the predicted fatigue lifespan of each binder, evaluated at strain amplitudes of 2.5% and 5%. These substantial deformation levels are pertinent in binder assessments, as the strain imparted to binders within pavements may reach fifty times that of the asphalt mixture as a whole [78]. The selection of 2.5% and 5% as reference points follows recommendations, with the former representing binder strain in thin pavement sections and the latter corresponding to pavements whose surface layers exceed 4.5 inches [79].

Figure 10 demonstrates that as strain amplitudes rise from 2.5% to 5%, the durability under repeated loading of both base asphalt and OUMM declines significantly, highlighting that pavement areas are more vulnerable to fatigue deterioration. At strain levels of 2.5% and 5%, asphalt binders modified with varying OUM concentrations demonstrated fatigue lives that were substantially greater than those observed for the unmodified one. When subjected to a 2.5% strain level, the fatigue life of OUMN mixtures at 2%, 4%, 6%, 8%, and 10% concentrations elevated by 34%, 77%, 97%, 149%, and 168% relative to the unmodified asphalt. For a 5% strain level, these mixtures exhibited improvements of 31%, 50%, 80%, 104%, and 136%, respectively. In this context, asphalt containing substantial OUM proportions is more appropriate for thicker pavement structures, whereas those with lower OUM concentrations are better suited for thinner pavement sections.

### 3.3. BBR Results

BBR test provides the S value and m-value, which are fundamental for characterizing asphalt performance in cold environments. S gauges the asphalt’s capacity to resist deformation, while m-value indicates its ability to dissipate internal stresses. Lower values of S are linked to greater ductility, reducing the likelihood of brittle failure due to temperature-induced contraction, whereas higher m-values denote improved stress relaxation. Thus, decreased S and increased m-value together signify enhanced resistance to deformation and stress, minimizing the occurrence of low-temperature cracking.

**Figure 11:**
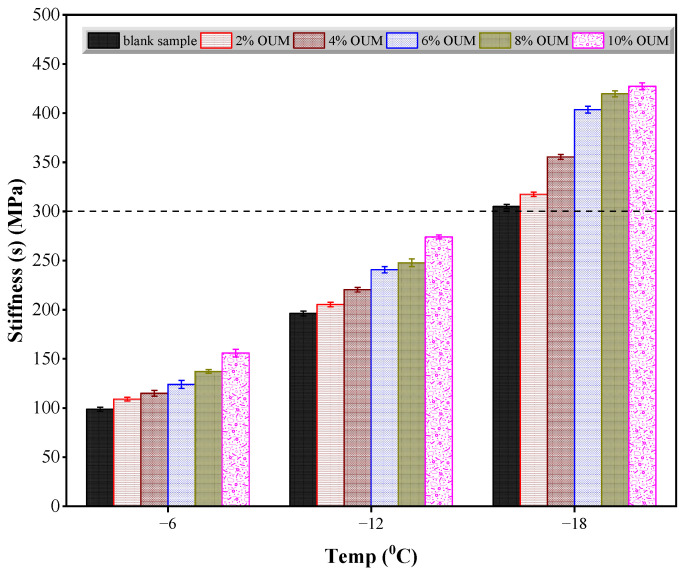
Results of creep stiffness.

**Figure 12:**
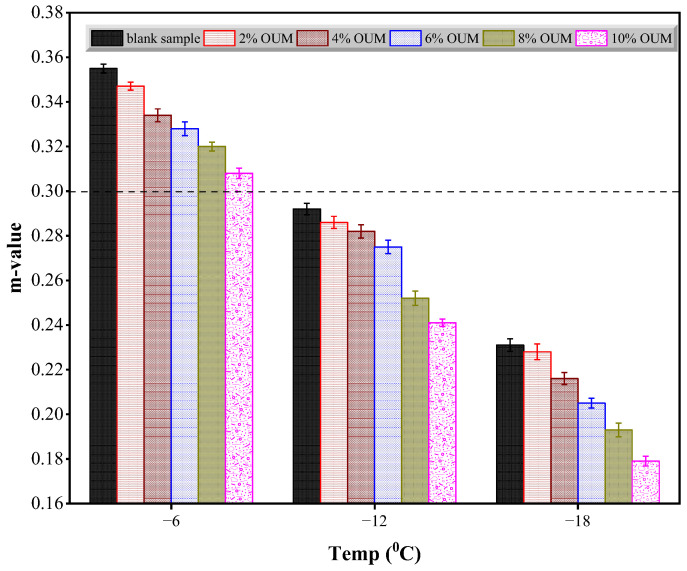
Results of creep rate.

According to Figure 11 and Figure 12, the bending beam rheometry analysis determined the S and m-values of unmodified and OUMM-containing samples at −6 °C, −12 °C, and −18 °C. Figure 11 reveals that a reduction in test temperature results in a steady rise in S values. Furthermore, at any given temperature, asphalts modified with OUM consistently display higher S values than the base binder. The data also show that increasing OUM content leads to higher S values, suggesting that OUM modifiers adversely affect asphalt’s low-temperature performance. As depicted in Figure 12, lowering the test temperature results in the m-value following a pattern contrary to S. At equivalent temperatures, OUMM samples demonstrate notably smaller m-values relative to unmodified asphalt. This reduction becomes more pronounced with a higher OUM percentage, highlighting the negative influence of OUM addition on the low-temperature capabilities of asphalt. Additionally, both the unmodified and OUMM samples comply with the Superpave specifications and specifically, an S-value of no more than 300 MPa and an m-value not less than 0.30 at −6 °C. However, neither sample satisfies these requirements at −12 °C or −18 °C. As represented in Figure 12, the m-values recorded at these lower temperatures fall below the 0.30 threshold. The detrimental effects are attributable to the OUM swelling, which decreases the proportion of light fractions in the binder, thereby weakening their resistance to deformation under low-temperature conditions.

### 3.4. FTIR Analysis

**Figure 13:**
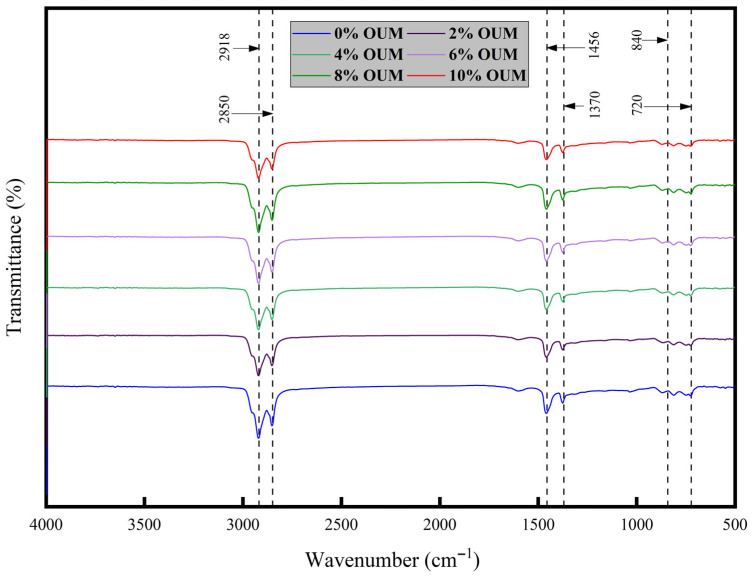
Obtained FTIR results.

The analysis of chemical functional groups within asphalt binders is generally accepted as a critical step in uncovering the underlying modification mechanism. Figure 13 presents the FTIR analysis results for unmodified asphalt alongside those of OUMM. Absorption bands appearing at 2918 cm^−1^ and 2850 cm^−1^ correspond to the antisymmetric and symmetric stretching vibrations of methylene (CH_2_) groups, respectively. Similarly, signals at 1456 cm^−1^ and 1370 cm^−1^ are attributed to the antisymmetric and symmetric bending vibrations of methyl (CH_3_) groups. Additionally, bands between 870 and 670 cm^−1^ are due to C-H vibrational modes that indicate substitution on the aromatic ring. As a result, saturated hydrocarbons and unsaturated compounds like aromatics, primarily composed of methyl and methylene groups, constitute the key components of unmodified and modified samples. The FTIR spectra obtained from all samples reveal consistent patterns, with the unmodified asphalt exhibiting identical chemical functional groups to those found in samples containing 2%, 4%, 6%, 8%, and 10% OUMM. The unmodified asphalt and OUMM share the same active chemical functionalities, and the major absorption bands across all asphalt binder samples are comparable. No clear indication of chemical reactions or new functional groups emerges after incorporating OUM into the control binder, suggesting that the modification is chiefly physical rather than chemical.

### 3.5. The Differences in Softening Point

**Figure 14:**
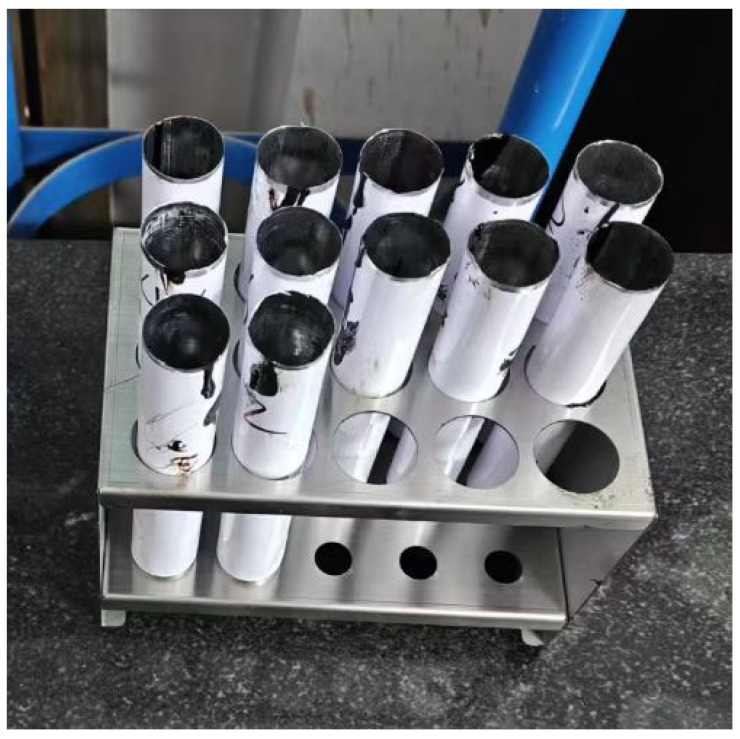
The aluminum tubes utilized in the test.

Significant variations in softening point measurements during controlled storage conditions serve as critical indicators of substantial segregation of modifying agents within the complex asphalt matrix, representing a fundamental challenge in polymer-modified asphalt technology. Figure 14 illustrates the aluminum tubes (140 mm length × 25 mm diameter) employed in the storage stability assessment protocol. Following the standardized separation test methodology, each tube was filled with 50 g of modified binder and subjected to thermal conditioning at 163 °C for 48 h under quiescent conditions, simulating extended storage scenarios encountered in field applications. Subsequently, rapid cooling at −4 °C for 4 h was applied to solidify the specimens. The tubes were then sectioned into three equal segments, with softening point measurements conducted on both top and bottom portions to quantify potential phase separation.

**Figure 15:**
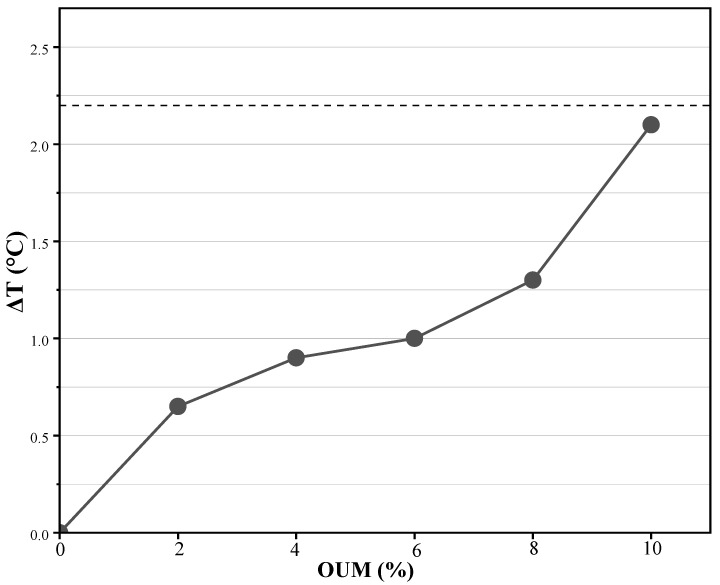
Effect of OUM on the storage stability.

Figure 15 displays the storage stability results for OUM materials under rigorously controlled testing conditions. The experimental data reveal that softening point variations (ΔT) in OUM demonstrate a consistent and notable increasing pattern that directly corresponds to higher OUM concentrations in the modified asphalt samples, suggesting a concentration-dependent degradation mechanism. This phenomenon intensified progressively with elevated OUM content in test specimens, creating increasingly larger disparities between upper and lower softening point measurements, indicating potential incompatibility issues at higher modifier loadings.

According to requirements, following standardized 48 h storage periods under specified environmental conditions including controlled temperature parameters, softening point variations must remain below the critical performance threshold of 2.2 °C [80,81] to ensure acceptable field performance characteristics and long-term durability in pavement applications. Comprehensive analysis of Figure 15 data confirms that ΔT measurements for modified asphalt incorporating systematically varied OUM concentrations of 2%, 4%, 6%, 8%, and 10% by total binder weight consistently stayed within acceptable parameters, demonstrating adequate storage stability across the entire tested range of modifier concentrations.

These findings have significant implications for quality control protocols in asphalt production facilities and suggest that OUM can be successfully incorporated into asphalt binders without compromising storage stability when used within the investigated concentration range. The observed trends indicate that while higher OUM concentrations may offer enhanced performance benefits, careful consideration must be given to storage stability requirements to ensure practical implementation success in commercial asphalt production environments.

## 4. Conclusions

The pursuit of sustainable and high-performance pavement materials has spurred significant interest in incorporating waste products into asphalt binders. This research addresses this by evaluating the efficacy of repurposing OUM waste as asphalt modifier through a series of laboratory investigations. The principal conclusions drawn from this study are listed as follows:

- The incorporation of 10% OUM by weight resulted in a seven-fold increase in the binder’s ZSV at 60 °C, indicating a marked improvement in its ability to resist permanent deformation (rutting).
- LAS tests revealed a dramatic improvement in fatigue resistance, with a 168% increase in fatigue life at a 2.5% strain amplitude for the 10% OUM-modified binder. The fatigue life parameter (A) increased significantly, indicating that the polymer network formed by OUM fibers is highly effective at resisting the initiation and propagation of fatigue cracks under repeated traffic loading.
- The significant gains in high-temperature and fatigue properties were offset by a decline in low-temperature performance. BBR tests showed a 55% increase in creep stiffness (S-value) and a 25% decrease in the m-value at −12 °C for the 10% OUM binder, indicating reduced relaxation capability and heightened susceptibility to thermal cracking.
- FTIR analysis suggested that the modification mechanism is primarily a physical process, involving the swelling and dispersion of PP to form a reinforcing network, rather than a chemical reaction. Importantly, modified binders containing up to 10% OUM demonstrated excellent phase stability, ensuring no separation issues during high-temperature storage and handling.
- Based on the balance of performance enhancement and material stability, a modification content of up to 10% OUM (by weight of asphalt) is considered a viable and effective concentration for achieving substantial performance gains without compromising the binder’s homogeneity. The enhanced high-temperature rheological properties indicate that OUM-modified binders are particularly well-suited for application in temperate and hot climates.
- The selection of an optimal OUM concentration is governed by a trade-off between performance, stability, and workability. The upper limit for this study was established at 10% OUM, as preliminary testing revealed that concentrations exceeding this value failed to meet storage stability criteria, indicating an unstable blend unsuitable for practical application. Within the stable 0–10% range, a clear recommendation depends on the intended application. For general use, a modifier content of 4–6% OUM is recommended, as it provides a substantial enhancement in rutting and fatigue resistance while maintaining a moderate impact on low-temperature performance and ensuring a high degree of stability. For specialized, high-stress applications in warmer climates, concentrations approaching the 10% limit may be considered to maximize high-temperature performance, but this must be weighed against the more significant reduction in low-temperature flexibility and the proximity to the stability threshold.

It is important to acknowledge the limitations of this study. The findings are based on laboratory evaluations using a single asphalt binder source and unutilized OUMs. Future research should validate these results with different binder types and actual post-consumer waste masks. Furthermore, a key future objective is to develop solutions for the complete utilization of OUMs, including the non-polymeric components, and to investigate hybrid modification approaches to mitigate the observed negative effects on low-temperature performance. Furthermore, while this study establishes the technical viability of OUM modification, future research should undertake a comprehensive life-cycle cost analysis to compare its economic feasibility against conventional polymer modifiers and quantify its overall environmental and economic impact.

The integration of OUM into the asphalt industry not only presents an innovative solution for a pressing environmental waste problem but also offers a practical method to enhance the durability and service life of pavement infrastructure.

## Figures and Tables

**Table 1:** Basic properties of the studied neat binder.

Property	Penetration	Ductility	Softening Point	Dynamic Viscosity	Flash Point	Penetration Index PI
	(25 °C/0.1 mm)	(15 °C/cm)	(°C)	(60 °C/Pa s)	(°C)	
Requirement	60–80	≥100	≥46	≥180	≥260	−1.5–+1.0
Result	71	>100	48	223	>300	−0.87

**Table 2:** Physical parameters characterizing OUM.

Test Item	Melting Point (°C)	Tensile Strength (MPa)	Water Absorption 24 h (%)	Specific Gravity	Rupture Force (N)
Result	155	3.45	8.2	0.91	19.38
Standard	ASTM-D7138-16	ASTM-D638-14	ASTM-D570-98	ASTM-D792-20	ASTM-D638-14

## Data Availability

The original contributions presented in this study are included in the article. Further inquiries can be directed to the corresponding authors.
